# Prokineticin 1–prokineticin receptor 1 signaling in trophoblast promotes embryo implantation and placenta development

**DOI:** 10.1038/s41598-021-93102-1

**Published:** 2021-07-02

**Authors:** Ewelina Goryszewska-Szczurek, Monika Baryla, Piotr Kaczynski, Agnieszka Waclawik

**Affiliations:** Institute of Animal Reproduction and Food Research, Polish Academy of Sciences, Tuwima 10, 10-748 Olsztyn, Poland

**Keywords:** Cell proliferation, Embryology, Intrauterine growth, Animal physiology

## Abstract

Successful pregnancy establishment in mammals depends on proper embryo-maternal communication. Prokineticin 1 (PROK1) is a secretory protein that exerts pleiotropic functions in various tissues. Despite the studies that have primarily been performed with human cell lines and mice, the function of PROK1 in trophoblasts has still not been fully elucidated. Hence, the aim of this study was to establish the role of PROK1 in trophoblasts during implantation and placentation. Prokineticin 1 mRNA was elevated in porcine trophoblasts during implantation and the early placentation period. Furthermore, we reveal that PROK1–PROKR1 signaling induces the expression of genes involved in the regulation of angiogenesis, immunological response, trophoblast cell adhesion, invasion, and proliferation, as well as stimulating phosphorylation of MAPK and PTK2. Ingenuity Pathway Analysis identified the aforementioned and also other functions associated with PROK1-regulated genes/proteins, such as cell-to-cell contact, epithelial tissue differentiation, Ca2+ release, lipid synthesis, and chemotaxis. We also showed evidence that PROK1 acting via PROKR1 increased trophoblast cell proliferation and adhesion. The PROK1-stimulated cell proliferation was mediated by PI3K/AKT/mTOR, MAPK, and cAMP, whereas adhesion was mediated by MAPK and/or PI3K/AKT signaling pathways. Concluding, our study suggests that PROK1 plays a pleiotropic role in trophoblast function during implantation and early placentation.

## Introduction

In all placental mammals, including pigs, the most important processes during early pregnancy are the development of endometrial receptivity, maternal recognition of pregnancy, implantation, and placentation. The maternal recognition of pregnancy, which in pigs is initiated on days 11–12 of pregnancy, is a process during which the conceptuses (embryos with associated membranes) signal their presence to the maternal system and prolong the lifespan of the corpus luteum (CL)^1^. At this time, porcine conceptuses secrete increased levels of estrogens (including estradiol-17β; one of the major porcine conceptus signals for the establishment of pregnancy) and undergo elongation from spherical forms to tubular forms and finally to filamentous forms^2^. This transformation allows for an increased contact surface between the conceptus trophoblast cells (trophectoderm) and uterine luminal epithelium (LE) which facilitates conceptus attachment to the endometrium. This, in turn, is the first and critical step for implantation in all mammalian species. In pigs, conceptus attachment occurs between days 14 and 19 of pregnancy^1^. Implantation involves pregnancy-specific interactions between integrins and extracellular matrix proteins (ECM) which contribute to stable adhesion to the endometrium^3,4^. Integrin binding in the LE and trophoblast tissue is mediated by secreted phosphoprotein 1 (SPP1/osteopontin)^5^.

Implantation and the establishment of pregnancy in pigs, as well as in other mammalian species, display hallmark signs of inflammation^3^. During this time, the expression and secretion of inflammatory mediators, such as interferon γ (IFNG) and δ (IFND), prostaglandins F2α (PGF2α) and E2 (PGE2), tumor necrosis factor α (TNF), interleukin 1B (IL1B), and cytokines from the interleukin 6 family, namely, interleukin 6 (IL6), interleukin 11 (IL11), and LIF interleukin 6 family cytokine (LIF), by the porcine endometrium and conceptus are increased^3,6^.

The implantation is followed by placentation. Pigs have a true epitheliochorial placenta in which the luminal epithelium remains intact throughout the entire pregnancy (i.e., non-invasive placentation). The main function of the placenta is the fetal–maternal exchange of gases, nutrients, hormones, cytokines and other regulatory molecules that support the growth and development of the fetus^1^. One of the most vital processes for placenta development is the formation of the local blood circulation system at the embryo–maternal interface, i.e., angiogenesis. Recent findings indicate that prokineticin 1 (PROK1) may serve as a novel angiogenic factor involved in the regulation of implantation and placentation^7–9^. This molecule (also called endocrine gland-derived VEGF) is a 11-kDa protein and its expression in humans has been reported, among others in the endometrium, placenta, trophoblast, ovary and fallopian tube^10^. Prokineticin 1 acts through two related receptors, namely, PROKR1 and PROKR2. Interestingly, in human trophoblast cells, *PROKR1* mRNA expression has been found to be 80 times higher when compared to the expression of *PROKR2* mRNA^11^. Besides angiogenesis, PROK1 may regulate a number of biological processes, including cell–cell contact, cell migration, proliferation, and inflammatory response^7,8,12^. Our recent study revealed that PROK1 could be an embryonic signal mediator which, acting via PROKR1, stimulates angiogenesis in the porcine endometrium during implantation and the early placentation period^7^. Moreover, PROK1 regulates the endometrial expression of pregnancy-related genes and proteins, as well as the prostaglandin and cytokine secretion in pigs^8^. In humans, the peak of PROK1 and PROKR1 expression occurs in placenta at 8–11 weeks of gestation and then gradually decreases by the end of the first-trimester of pregnancy^11^. Interestingly, impaired PROK1 signaling has been linked with placental pathologies such as preeclampsia and intrauterine growth restriction^12^.

Studies on PROK1 function during implantation and placentation have mainly been performed on human cell lines (e.g., HTR-8/SVneo or BeWo cells) and rarely with ex vivo placenta tissues^13,14^ or mice^15^. Thus, the function of PROK1 in conceptuses/trophoblasts during early pregnancy has not been fully elucidated yet. Based on our recent data and previous studies, we hypothesize that during early pregnancy, PROK1, acting through its receptor, contributes to trophoblast attachment/implantation and early placentation. Therefore, the objectives of the present study were the following: (1) to determine the gene expression profiles of *PROK1* and *PROKR1* in porcine conceptuses/trophoblasts during early pregnancy; (2) to study the expression profiles of pregnancy-related genes such as *SPP1,* biglycan (*BGN)*, fibroblast growth factor 9 (*FGF9*), and *IL11* in porcine trophoblasts; (3) to identify pregnancy-related genes activated by PROK1–PROKR1 interaction in trophoblasts; and (4) to elucidate the effect of PROK1 on porcine trophoblast cell proliferation and adhesion to ECM proteins and to identify the signaling pathways involved in these processes.

## Results

### The gene expression of prokineticin 1 and some of pregnancy-related genes is upregulated in the conceptus/trophoblast during embryo implantation and early placentation (experiment 1)

**Figure 1:**
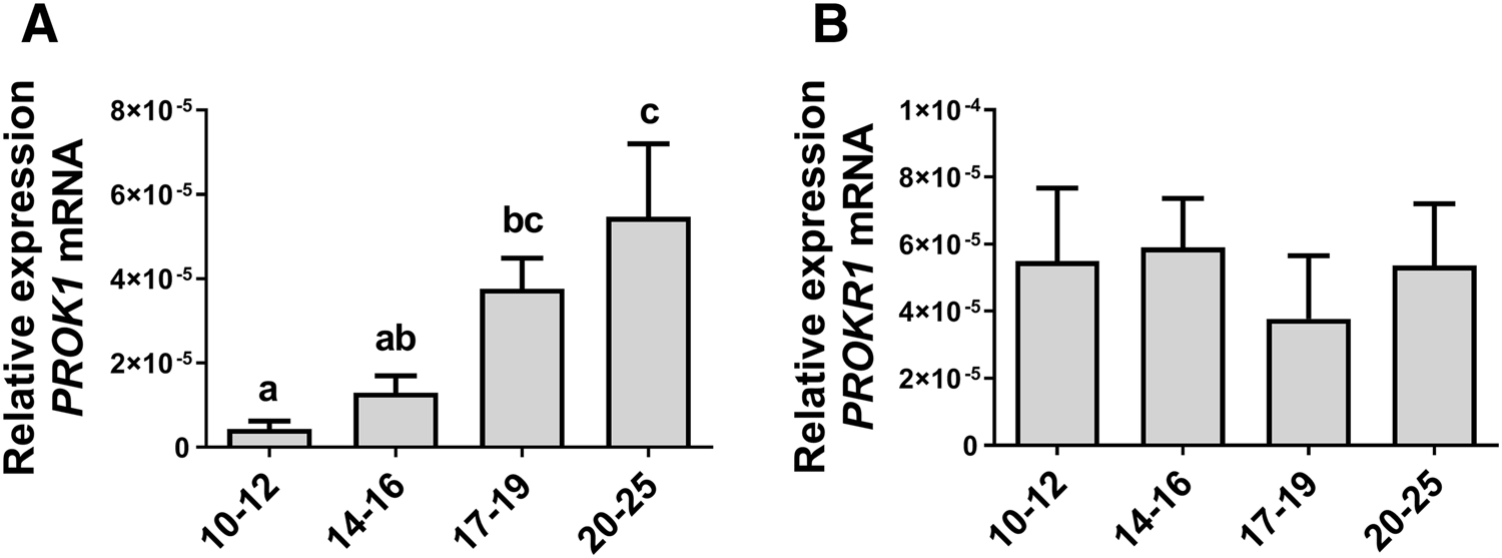
Expression of prokineticin 1 (*PROK1*) mRNA is upregulated in the conceptus/trophoblast during embryo implantation and early placentation. The expression profiles of prokineticin 1 (*PROK1*) (**A**) and prokineticin receptor 1 (*PROKR1*) (**B**) mRNA in the porcine conceptus/trophoblast on days 10–12 (preimplantation stage; spherical and tubular forms), 14–16 (beginning of attachment/implantation; filamentous forms), 17–19 (implantation stage, filamentous forms) and 20–25 (chorion which originates from trophoblast, the onset of placenta development) of pregnancy. Data are presented as means ± SEM. Different letters (a–c) indicate statistically significant differences (*P* < 0.05). Means sharing at least one the same letter indicate no significant difference.

To determine the expression profile of *PROK1*, *PROKR1*, *BGN*, *IL11*, and *SPP1* mRNA we used conceptus/trophoblast tissue collected from pregnant gilts between 10 and 25 days of gestation. The expression of the *PROK1* gene in the conceptus (days 10–17) and trophoblast (days 18–25) increased gradually in the studied stages of pregnancy. Trophoblast expression of *PROK1* mRNA was significantly upregulated on days 17–19 (implantation stage) compared to days 10–12 (preimplantation stage) of pregnancy. The abundance of *PROK1* mRNA reached the highest levels on days 20–25 of pregnancy (the onset of placenta development) compared to earlier stages (attachment/implantation; days 10–12, and 14–16 of pregnancy; *P* < 0.05; Fig. 1A). No changes in the expression of *PROKR1* mRNA in the trophoblast were observed during studied stages of pregnancy (*P* > 0.05; Fig. 1B).

**Figure 2:**
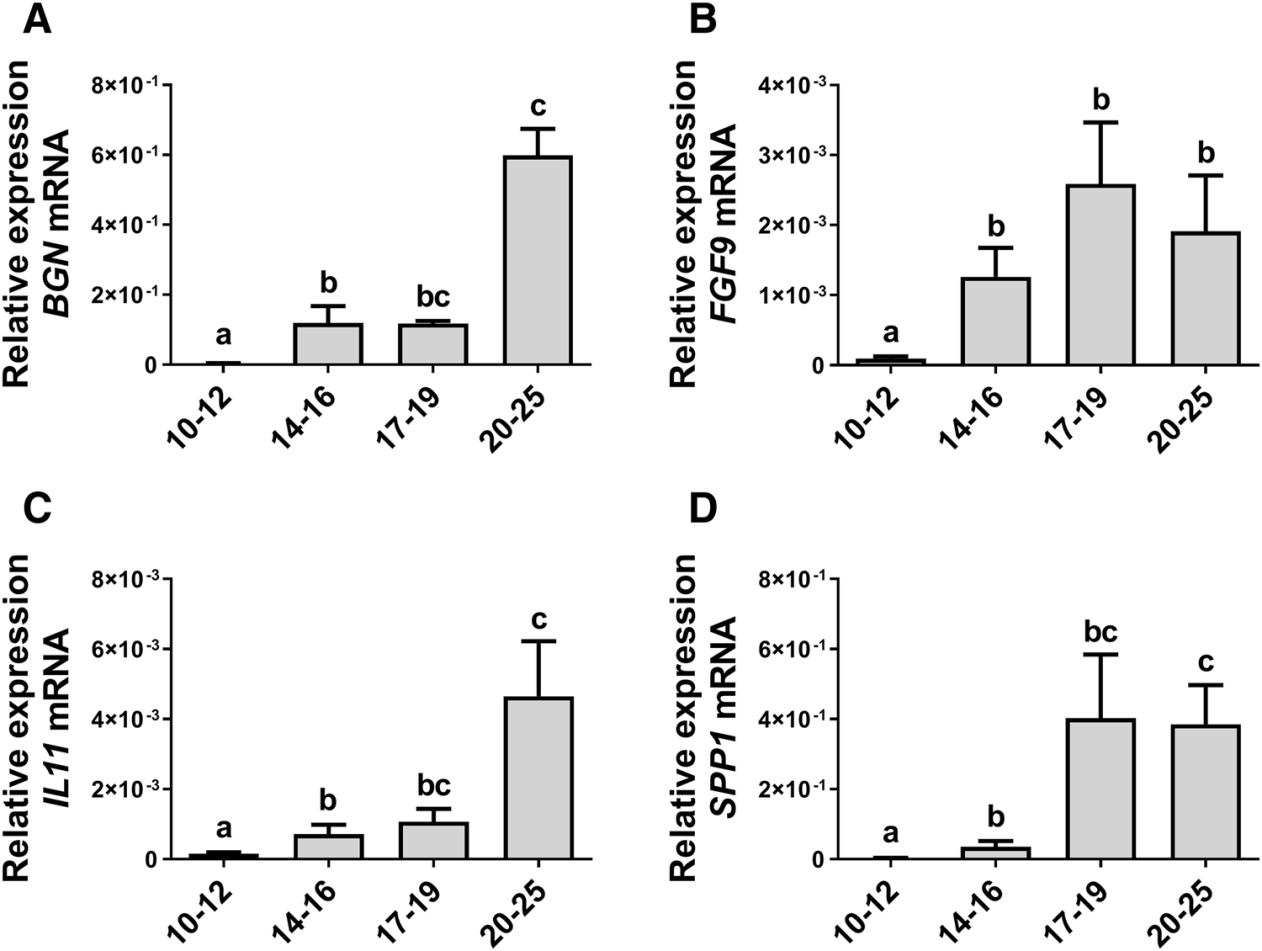
Expression of some of pregnancy-related genes is upregulated in the conceptus/trophoblast during embryo implantation and early placentation. The expression profiles of biglycan (*BGN*) (**A**); fibroblast growth factor 9 (*FGF9*) (**B**); interleukin 11 (*IL11*) (**C**), and secreted phosphoprotein 1 (*SPP1*) (**D**) mRNA in the porcine conceptus/trophoblast on days 10–12 (preimplantation stage; spherical and tubular forms), 14–16 (beginning of attachment/implantation; filamentous forms), 17–19 (implantation stage, filamentous forms) and 20–25 (chorion which originates from trophoblast, the onset of placenta development) of pregnancy. Data are presented as means ± SEM. Different letters (a–c) indicate statistically significant differences (*P* < 0.05). Means sharing at least one the same letter indicate no significant difference.

The expression of *BGN*, *IL11*, and *SPP1* mRNA in the conceptus/trophoblast increased gradually in the studied stages of pregnancy (days 10–25). Trophoblast expression of these genes was significantly upregulated on days 14–16, and 17–19, compared to days 10–12 of pregnancy and reached the highest levels on days 20–25 of pregnancy (*P* < 0.05; Fig. 2A,C,D). The expression of *FGF9* mRNA was significantly increased on days 14–16, 17–19, and 20–25, compared to days 10–12 of pregnancy (*P* < 0.05; Fig. 2B).

### The expression of the pregnancy-related genes is stimulated by PROK1 in the trophoblast in vitro (experiment 2)

To investigate the effect of PROK1 on the expression of pregnancy-related genes we used an in vitro model of porcine trophoblast cells collected on days 15 and 20 of pregnancy. Primary trophoblast cells were incubated with PROK1 (40 nM) for 24 h in the presence/absence of a PROKR1 antagonist (PC7; 1 µM).

#### PROK1 increases the expression of the genes involved in angiogenesis in the porcine trophoblast

**Figure 3:**
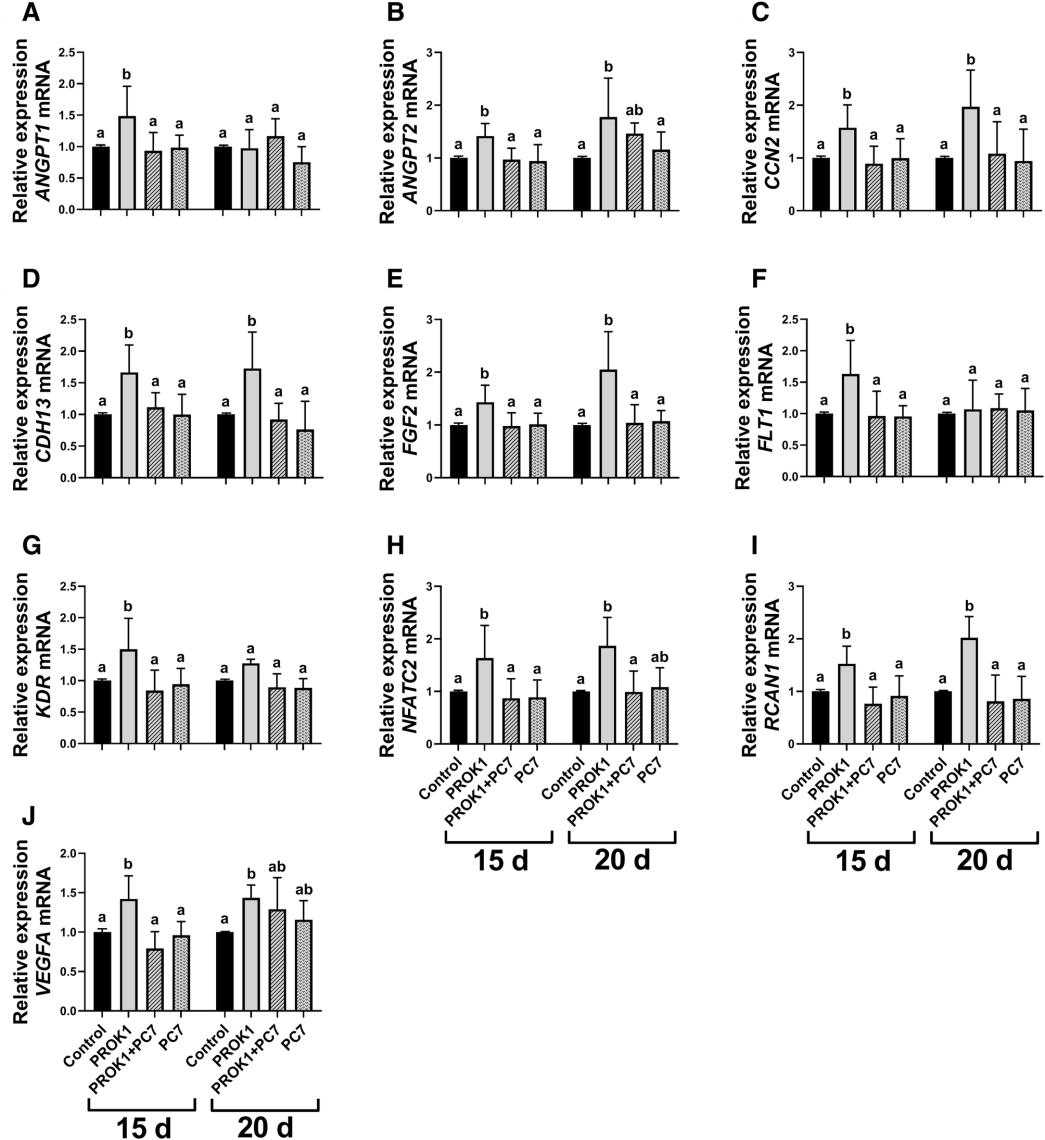
Expression of the genes involved in angiogenesis in the porcine trophoblast is stimulated by the prokineticin 1 (PROK1). The effect of PROK1 on the expression of genes involved mainly in angiogenesis in the porcine trophoblast on days 15 and 20 of pregnancy in vitro: (**A**) angiopoietin 1 (*ANGPT1*); (**B**) angiopoietin 2 (*ANGPT2*); (**C**) cellular communication network factor 2 (*CCN2*); (**D**) cadherin 13 (*CDH13*); (**E**) fibroblast growth factor 2 (*FGF2*); (**F**) fms-related receptor tyrosine kinase 1 (*FLT1*); (**G**) kinase insert domain receptor (*KDR*); (**H**) nuclear factor of activated T cells 2 (*NFATC2*); (**I**) regulator of calcineurin 1 (*RCAN1*), and (**J**) vascular endothelial growth factor A (*VEGFA*). Primary trophoblast cells were incubated with the control (vehicle) or PROK1 (40 nM) for 24 h in the presence/absence of a PROKR1 antagonist (PC7; 1 µM). Data are presented as the mean ± SEM of the fold change versus control. Different letters (a–b) indicate statistically significant differences (*P* < 0.05). Means sharing at least one the same letter indicate no significant difference.

Studying the influence of PROK1 on the expression of angiogenesis-related genes in the porcine trophoblast in vitro, we found an effect of treatment, an effect of day of pregnancy, and a treatment x day of pregnancy interaction for the expression of fibroblast growth factor 2 (*FGF2*) and vascular endothelial growth factor A (*VEGFA*) genes (*P* < 0.05; Fig. 3E,J, respectively). A significant effect of treatment and treatment × day of pregnancy interaction was detected for the expression of angiopoetin-1 (*ANGPT1*) gene (*P* < 0.05, Fig. 3A). A significant effect of treatment and an effect of pregnancy stage were detected for the expression of angiopoetin-2 (*ANGPT2*) gene (*P* < 0.05, Fig. 3B). Furthermore, a significant effect of treatment was detected for the expression of cellular communication network factor 2 (*CCN2*), cadherin 13 (*CDH13*), fms-related receptor tyrosine kinase 1 (*FLT1*; also known as VEGF receptor 1), kinase insert domain receptor (*KDR*; also known as VEGF receptor 2), nuclear factor of activated T cells 2 (*NFATC2*) and regulator of calcineurin 1 (*RCAN1*) genes (*P* < 0.05; Fig. 3C,D and F–I, respectively).

We found that 24 h-treatment with PROK1 stimulated the trophoblast expression of genes involved in angiogenesis: *ANGPT1*, *FLT1* and *KDR* on day 15 of pregnancy (*P* < 0.05; Fig. 3A,F,G, respectively) and *ANGPT2, CCN2*, *CDH13*, *FGF2*, *NFATC2*, *RCAN1*, and *VEGFA* (*P* < 0.05; Fig. 3B–E, and H–J, respectively) on days 15 and 20 of pregnancy. These stimulating effects of PROK1 on gene expression were abolished by using a prokineticin receptor 1 antagonist (PC7) for all genes, except for *ANGPT2* and *VEGFA* in trophoblast cells isolated from trophoblasts on day 20 of pregnancy.

#### PROK1 increases the expression of the genes involved in immunological response in the porcine trophoblast

**Figure 4:**
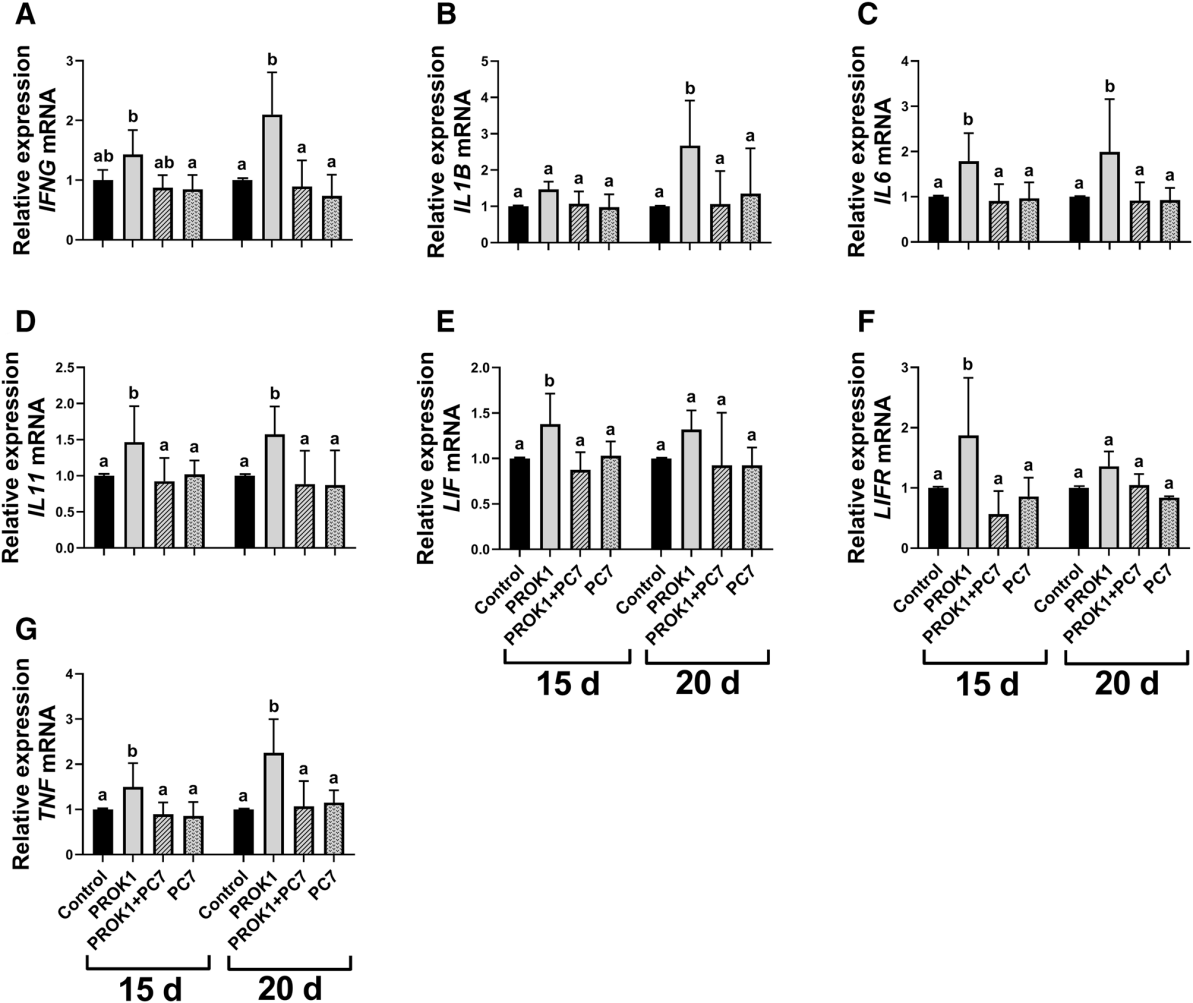
Expression of the genes involved in involved in immunological response in the porcine trophoblast is stimulated by the prokineticin 1 (PROK1). The effect of PROK1 on the expression of genes involved mainly in the immunological response in the porcine trophoblast on days 15 and 20 of pregnancy in vitro: (**A**) interferon gamma (*IFNG*); (**B**) interleukin 1 beta (*IL1B*); (**C**) interleukin 6 (*IL6*); (**D**) interleukin 11 (*IL11*); (**E**) LIF interleukin 6 family cytokine (*LIF*); (**F**) LIF receptor subunit alpha (*LIFR*), and (**G**) tumor necrosis factor (*TNF*). Primary trophoblast cells were incubated with the control (vehicle) or PROK1 (40 nM) for 24 h in the presence/absence of a PROKR1 antagonist (PC7; 1 µM). Data are presented as the mean ± SEM of the fold change versus control. Different letters (a–b) indicate statistically significant differences (*P* < 0.05). Means sharing at least one the same letter indicate no significant difference.

Using the in vitro model of trophoblast cells, we found an effect of treatment, and a treatment × day of pregnancy interaction for the expression of *IFNG* gene (*P* < 0.05; Fig. 4A). A significant effect of treatment and an effect of day of pregnancy were detected for the expression of *IL1B* gene (P < 0.05; Fig. 4B). Moreover, a significant effect of treatment was detected for the expression of *IL6*, *IL11*, *LIF*, LIF receptor subunit alpha (*LIFR*), and *TNF* genes (*P* < 0.05, Fig. 4C–G, respectively).

Elevated expression of genes involved in immunological response after PROK1 treatment was observed in trophoblast cells for *LIF* and *LIFR* on day 15 of pregnancy (*P* < 0.05; Fig. 4E,F, respectively); *IFNG* and *IL1B* on day 20 of pregnancy (*P* < 0.05; Fig. 4A,B, respectively) and *IL6*, *IL11*, and *TNF* on both days 15 and 20 of pregnancy (*P* < 0.05; Fig. 4C,D,G, respectively). Stimulating effects of PROK1 on the gene expression were abolished by using PC7 for all genes.

#### PROK1 increases the expression of the genes involved in trophoblast proliferation, adhesion and the regulation of trophoblast invasion

**Figure 5:**
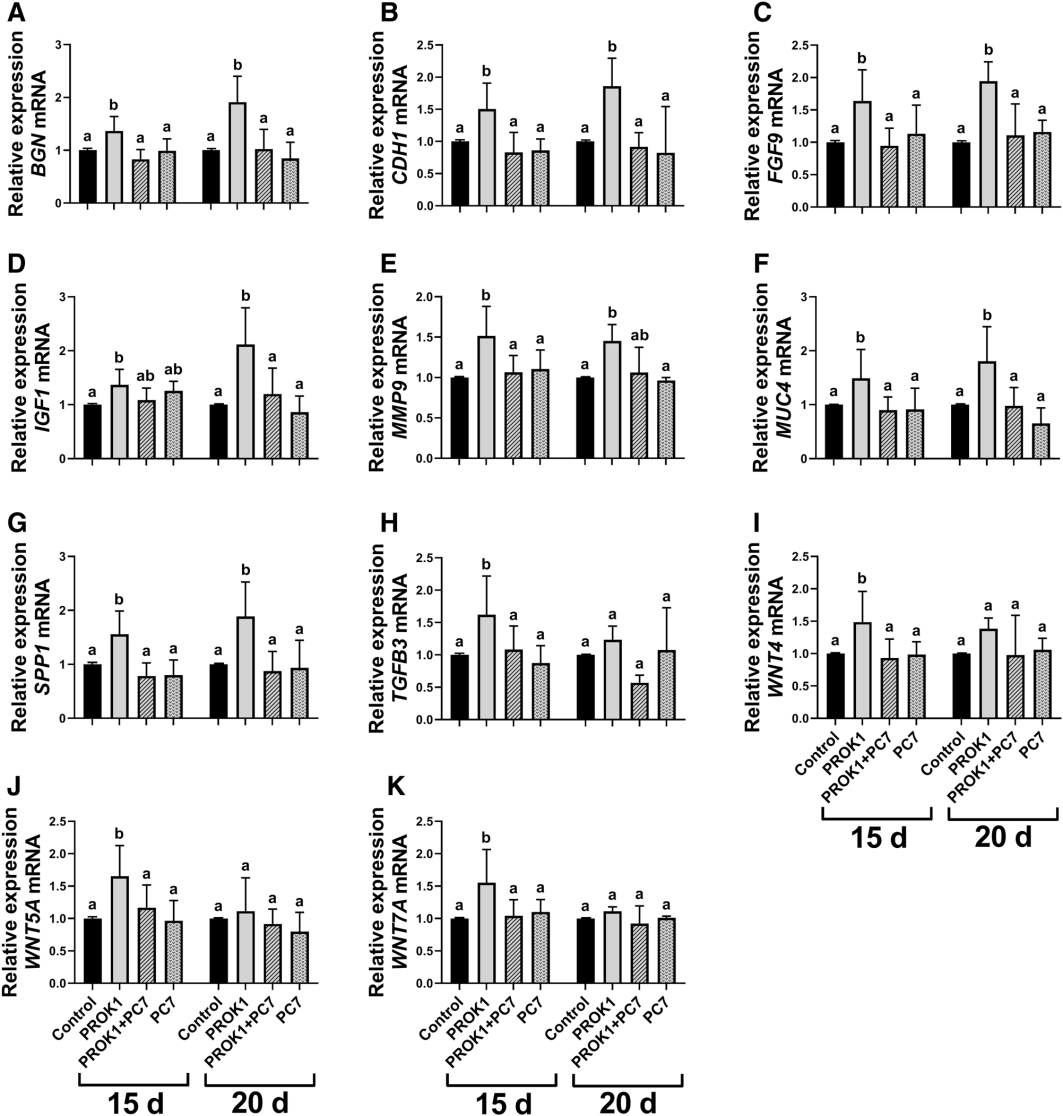
Expression of the genes involved in the regulation of proliferation, adhesion and invasion in the porcine trophoblast is stimulated by the prokineticin 1 (PROK1). The effect of PROK1 on the expression of genes involved mainly in the regulation of proliferation, adhesion and invasion in the porcine trophoblast cells on days 15 and 20 of pregnancy in vitro: (**A**) biglycan (*BGN*); (**B**) cadherin 1 (*CDH1*); (**C**) fibroblast growth factor 9 (*FGF9*); (**D**) insulin-like growth factor 1 (*IGF1*); (**E**) matrix metallopeptidase 9 (*MMP9*); (**F**) mucin 4 (*MUC4*); (**G**) secreted phosphoprotein 1 (*SPP1*); (**H**) transforming growth factor beta 3 (*TGFB3*); (**I**) Wnt family member 4 (*WNT4*); (**J**) Wnt family member 5A (*WNT5A*), and (**K**) Wnt family member 7A (*WNT7A*). Primary trophoblast cells were incubated with the control (vehicle) or PROK1 (40 nM) for 24 h in the presence/absence of a PROKR1 antagonist (PC7; 1 µM). Data are presented as the mean ± SEM of the fold change versus control. Different letters (a–b) indicate statistically significant differences (*P* < 0.05). Means sharing at least one the same letter indicate no significant difference.

Studying the influence of PROK1 on the expression of genes involved in proliferation, adhesion and invasion in the porcine trophoblast in vitro, we found an effect of treatment, and a treatment x day of pregnancy interaction for the expression of *BGN* and insulin-like growth factor 1 (*IGF1*) gene (*P* < 0.05; Fig. 5A,D). A significant effect of treatment and an effect of day of pregnancy were detected for the expression of Wnt family member 5A (*WNT5A*) and Wnt family member 7A (*WNT7A*) genes (*P* < 0.05; Fig. 5J,K, respectively). Furthermore, a significant effect of treatment was detected for the expression of cadherin 1 (*CDH1*), *FGF9*, matrix metallopeptidase 9 (*MMP9*), mucin 4 (*MUC4*), *SPP1*, transforming growth factor beta 3 (*TGFB3*), and Wnt family member 4 (*WNT4*) genes (*P* < 0.05; Fig. 5B,C and E–I, respectively).

Treatment of the trophoblast cells with PROK1 for 24 h increased the expression of *TGFB3*, *WNT4*, *WNT5A*, and *WNT7A* mRNA on day 15 of pregnancy (*P* < 0.05; Fig. 5H–K, respectively) and *BGN*, *CDH1*, *FGF9*, *IGF1 MMP9*, *MUC4*, and *SPP1* mRNA on days 15 and 20 of pregnancy (*P* < 0.05; Fig. 5A–G, respectively). These effects were abolished by using PC7 for all genes, except for *IGF1* on day 15 of pregnancy and *MMP9* in trophoblast cells isolated from conceptuses on day 20 of pregnancy.

### PROK1 promotes trophoblast cell proliferation and adhesion and activates signaling pathways involved in these processes (experiment 3)

#### PROK1 induces phosphorylation of MAPK1/3 and PTK2

**Figure 6:**
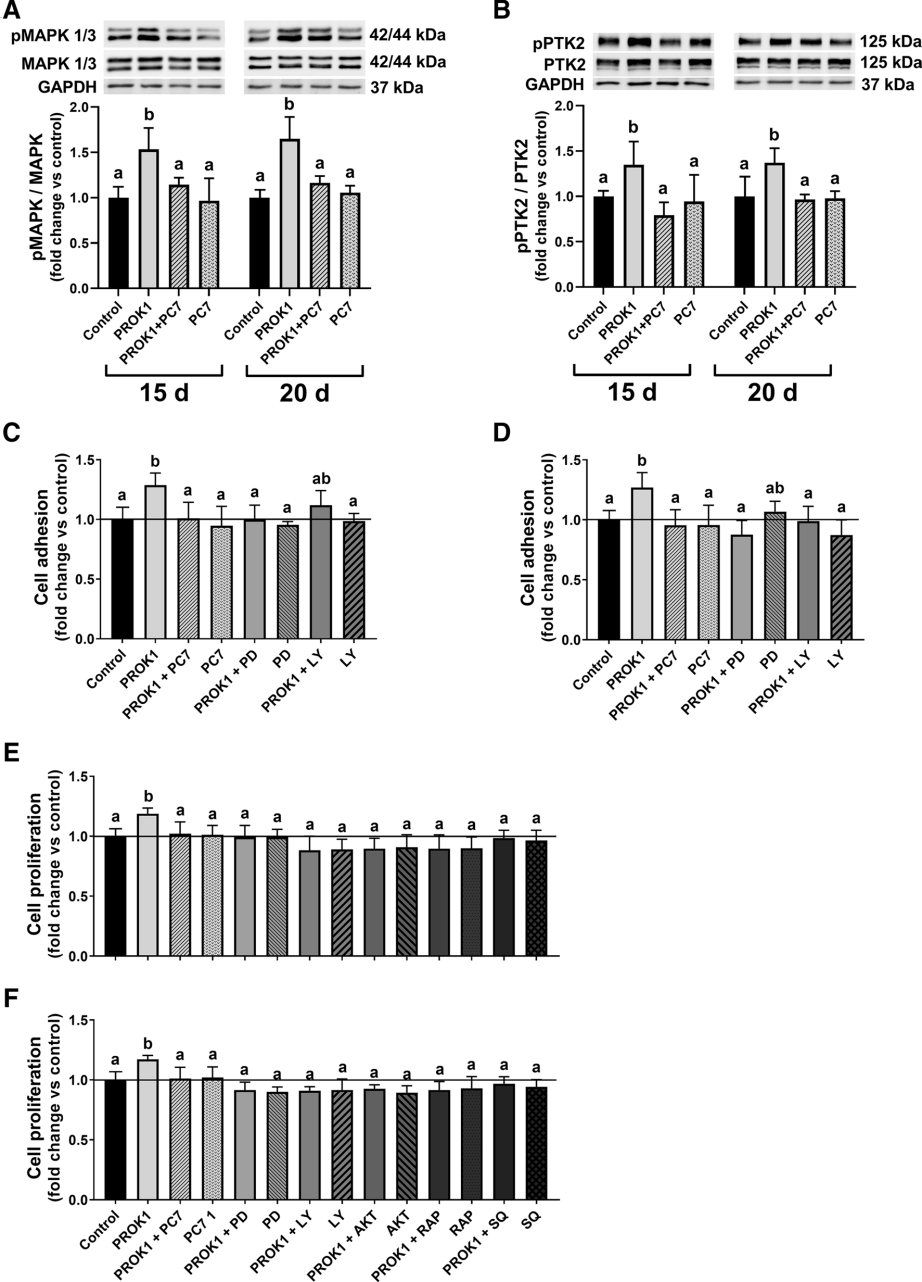
Prokineticin 1 (PROK1) stimulated trophoblast cell proliferation and adhesion and activates signaling pathways involved in these processes. The effects of PROK1 on processes involved in the porcine trophoblast cell proliferation, adhesion and invasion. PROK1 increases phosphorylation of mitogen-activated protein kinase (MAPK1/3) (**A**) and protein tyrosine kinase 2 (PTK2) (**B**) in trophoblast cells after 10 min of treatment in vitro. Representative samples of Western blots for MAPK1/3, pMAPK1/3, PTK2, and pPTK2 are shown in the upper part of (**A**) and (**B**). Full-length Western blots are presented in Supplementary Figs. 1 and 2. PROK1 stimulates adhesion and proliferation of trophoblast cells isolated on days 15 (**C**,**E**, respectively) and 20 (D and F, respectively) of pregnancy. Trophoblast cells were incubated for 24 h with the control (vehicle; 0.01% ethanol) or PROK1 in the presence/absence of a PROKR1 antagonist (PC7; 1 μM) and inhibitors of MAPK (PD 098059; 25 μM), phosphoinositide 3-kinase (PI3K; LY 294002; 25 μM)—for adhesion and proliferation assay; and inhibitors of protein kinase B (AKT; 2.5 μM), mTOR (rapamycin, RAP; 50 nM), and cAMP synthesis (SQ 22536; 25 μM)—only for proliferation assay. Data are presented as the mean ± SEM of the fold change versus control. Different letters (a–b) indicate statistically significant differences (*P* < 0.05). Means marked by the same letter indicate no significant difference.

To determine the effect of PROK1 on the mitogen-activated protein kinase (MAPK1/3) and protein tyrosine kinase 2 (PTK2; also known as FAK) phosphorylation we used an in vitro model of porcine trophoblast cells. Primary trophoblast cells collected on days 15 and 20 of pregnancy were incubated with the PROK1 (40 nM) for 10 min in the presence/absence of a PROKR1 antagonist (PC7; 1 µM). Phosphorylation of MAPK1/3 and PTK2 in porcine trophoblast cells was affected by treatment with PROK1 (*P* < 0.05) but not by the day of pregnancy on which the cells were collected. PROK1 induced the phosphorylation of MAPK1/3 and PTK2 proteins in trophoblast cells collected on days 15 and 20 of pregnancy (*P* < 0.05; Fig. 6A,B, respectively). These effects were abolished by using the PROK1 receptor antagonist PC7.

#### PROK1 stimulates porcine trophoblast cell adhesion and proliferation

To test whether PROK1 has an effect on adhesion and proliferation of trophoblast cells and to study the cellular pathways involved in these processes, we used in vitro models of porcine trophoblast cells from days 15 and 20 of pregnancy. Primary trophoblast cells were incubated with the PROK1 (40 nM) for 24 h in the presence/absence of a PROKR1 antagonist (PC7; 1 µM) and inhibitors of MAPK (PD 098059; 25 μM), phosphoinositide 3-kinase (PI3K; LY 294002; 25 μM)—for adhesion and proliferation assay; and inhibitors of protein kinase B (AKT; 2.5 μM), mammalian target of rapamycin (mTOR; rapamycin; 50 nM), and cAMP synthesis (SQ 22536; 25 μM)—only for proliferation assay. PROK1 after 24 h of incubation increased the adhesion to fibronectin by the trophoblast cells collected on days 15 and 20 of pregnancy (*P* < 0.05; Fig. 6C,D, respectively). The stimulating effects of PROK1 on trophoblast cell adhesion on days 15 (Fig. 6C) and 20 of pregnancy (Fig. 6D) were abolished by using PC7. Moreover, using inhibitors of MAPK (on days 15 and 20 of pregnancy) and PI3K (on day 20 of pregnancy) significantly reduced PROK1-stimulated adhesion in trophoblast cells.

We also showed that proliferation of porcine trophoblast cells collected from gilts on days 15 and 20 of pregnancy was significantly stimulated by PROK1 (*P* < 0.05; Fig. 6E,F, respectively) after 24 h of incubation. The stimulating effects of PROK1 on trophoblast cell proliferation on days 15 (Fig. 6E) and 20 of pregnancy (Fig. 6F) were inhibited by using PC7. Likewise, using inhibitors of MAPK, PI3K, AKT kinase, mTOR kinase, and cAMP synthesis significantly reduced PROK1-stimulated proliferation in trophoblast cells on days 15 and 20 of pregnancy.

### Identifications of biofunctions enriched for PROK1-regulated genes/proteins in the porcine trophoblast (experiment 4)

**Figure 7:**
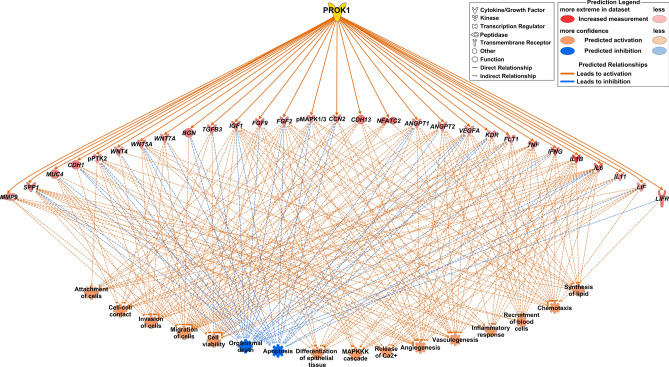
Putative pleiotropic role of prokineticin 1 (PROK1) in the porcine trophoblast during the establishment of pregnancy. The IPA database was used to evaluate the functions of genes up-regulated and proteins activated by PROK1 (vehicle versus PROK1) in the porcine trophoblast collected on days 15 and 20 of pregnancy. The predicted activation is based on an absolute z-score > 2 and the *P* value of the gene overlap. The icons and color scheme are IPA defaults. Angiopoietin 2 (*ANGPT2*); cellular communication network factor 2 (*CCN2*); cadherin 13 (*CDH13*); fibroblast growth factor 2 (*FGF2*); fms-related receptor tyrosine kinase 1 (*FLT1*); kinase insert domain receptor (*KDR*); nuclear factor of activated T cells 2 (*NFATC2*); vascular endothelial growth factor A (*VEGFA*); phosphorylated mitogen-activated protein kinase 1/3 (pMAPK1/3); phosphorylated protein tyrosine kinase 2 (pPTK2); interferon gamma (*IFNG*); interleukin 1 beta (*IL1B*); interleukin 6 (*IL6*); interleukin 11 (*IL11*); LIF interleukin 6 family cytokine (*LIF*); LIF receptor subunit alpha (*LIFR*); tumor necrosis factor (*TNF*); biglycan (*BGN*); cadherin 1 (*CDH1*); fibroblast growth factor 9 (*FGF9*); insulin-like growth factor 1 (*IGF1*); matrix metallopeptidase 9 (*MMP9*); mucin 4 (*MUC4*); secreted phosphoprotein 1 (*SPP1*); transforming growth factor beta 3 (*TGFB3*); Wnt family member 4 (*WNT4*); Wnt family member 5A (*WNT5A*); Wnt family member 7A (*WNT7A*).

Although the function of genes or protein selected for expression studies has been already known it is difficult to make a clear conclusion about the involvement of PROK1 in processes in which PROK1-regulated genes are involved. It is related to fact that some genes may be involved in stimulation whereas other genes—in inhibition of particular process. Thus, to minimize the bias the whole gene set was analyzed using Ingenuity Pathway Analysis (IPA) software to investigate the processes potentially related to PROK1 action. Functional annotation analyses performed using IPA revealed biological function categories with 4 or more molecules/genes significantly regulated by PROK1 (*P* < 1.8 × 10^–11^) such as: cell attachment, migration and invasion, cell–cell contact, cell viability, organismal death, apoptosis, the differentiation of epithelial tissue, MAPKKK cascade, release of Ca2+, angiogenesis, vasculogenesis, inflammatory response, the recruitment of blood cells, chemotaxis, and the synthesis of lipids (Fig. 7). Results were also summarized in tabular format (Supplementary Table 3) which contains identified terms, enrichment *P* value and activation/inhibition prediction parameter (Z-score)^16^.

## Discussion

**Figure 8:**
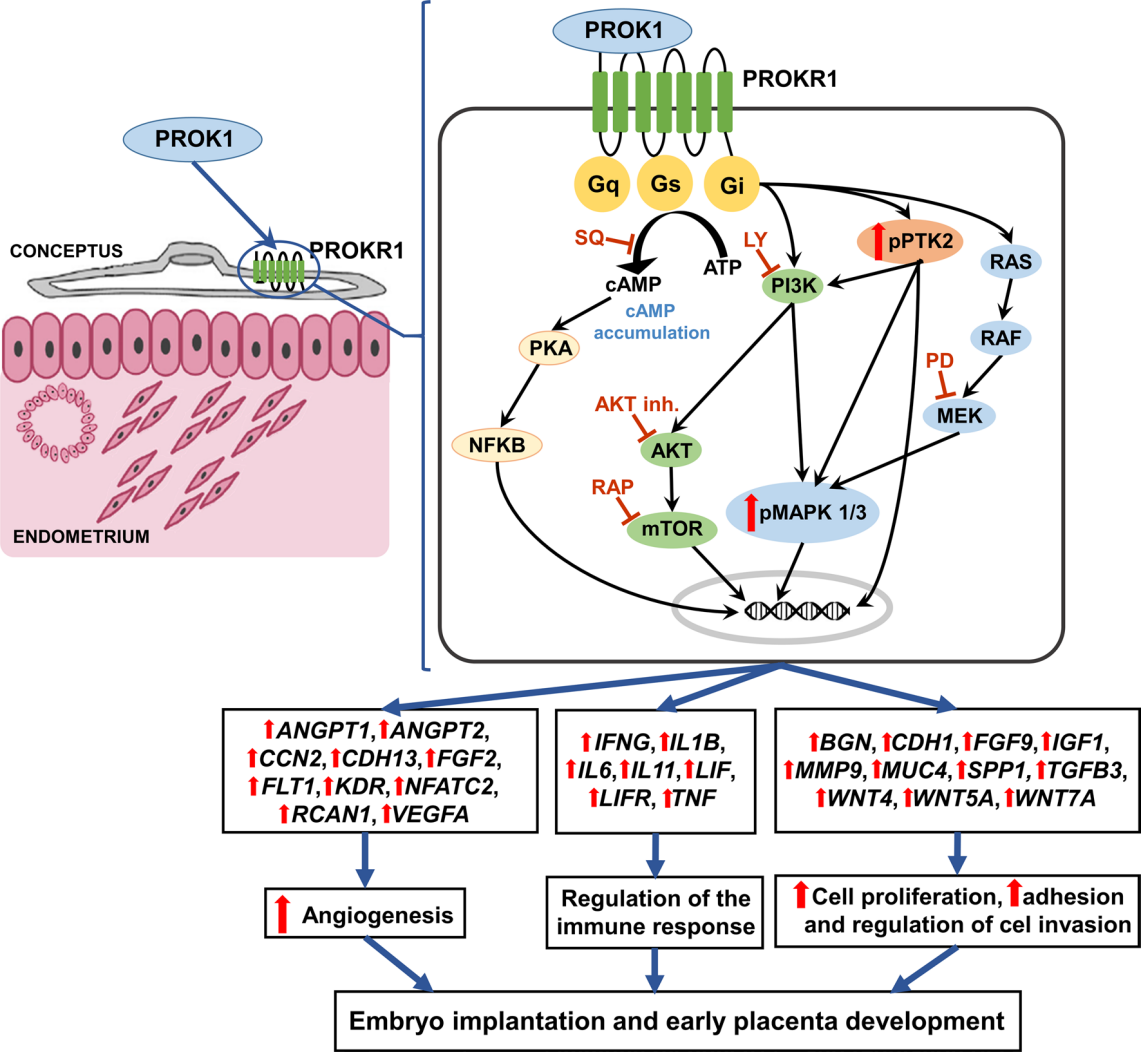
Proposed mechanism of prokineticin 1 (PROK1) contribution to conceptus/trophoblast attachment and early placentation in pigs. A greater abundance of PROK1, acting through PROK1 receptor (PROKR1), promotes primary trophoblast cell proliferation and adhesion. The PROK1-stimulated proliferation of trophoblast cells on days 15 and 20 of pregnancy is mediated by PROKR1 and occurs via phosphoinositide 3-kinase (PI3K), protein kinase B (AKT), mTOR kinase (mTOR) and phosphorylated mitogen-activated protein kinase (MAPK) signaling pathways, and accumulation of cyclic adenosine monophosphate (cAMP). The PROK1-induced adhesion of trophoblast cells is mediated by MAPK (on days 15 and 20 of pregnancy) and PI3K/AKT signaling pathways (on day 20 of pregnancy). Specific inhibitors used for MAPK (PD098059; PD), PI3K (LY294002; LY), AKT1/2 and mTOR (rapamycin, RAP) kinases, as well as for cAMP (SQ22536; SQ) signaling suppress the PROK1-induced proliferation of trophoblast cells. Specific inhibitors used for kinases of MAPK and PI3K inhibit PROK1-induced adhesion of trophoblast cells. PROK1 acting by PROKR1 regulates the expression of the pregnancy-related genes which are involved in processes such as angiogenesis, immunological response, trophoblast cell adhesion, invasion and proliferation. G-proteins q, s and i (Gq, Gs, Gi), RAS protein (RAS), RAF kinase (RAF), protein kinase A (PKA), the nuclear factor kappa-light-chain-enhancer of activated B cell (NFKB), adenosine triphosphate (ATP), phosphorylated mitogen-activated protein kinase 1/3 (pMAPK1/3); phosphorylated protein tyrosine kinase 2 (pPTK2); angiopoietin 1 (*ANGPT1*); angiopoietin 2 (*ANGPT2*); cellular communication network factor 2 (*CCN2*); cadherin 13 (*CDH13*); fibroblast growth factor 2 (*FGF2*); fms-related receptor tyrosine kinase 1 (*FLT1*); kinase insert domain receptor (*KDR*); nuclear factor of activated T cells 2 (*NFATC2*); regulator of calcineurin 1 (*RCAN1*); vascular endothelial growth factor A (*VEGFA*); interferon gamma (*IFNG*); interleukin 1 beta (*IL1B*); interleukin 6 (*IL6*); interleukin 11 (*IL11*); LIF interleukin 6 family cytokine (*LIF*); LIF receptor subunit alpha (*LIFR*); tumor necrosis factor (*TNF*); biglycan (*BGN*); cadherin 1 (*CDH1*); fibroblast growth factor 9 (*FGF9*); insulin-like growth factor 1 (*IGF1*); matrix metallopeptidase 9 (*MMP9*); mucin 4 (*MUC4*); secreted phosphoprotein 1 (*SPP1*); transforming growth factor beta 3 (*TGFB3*); Wnt family members (*WNT4*, *WNT5A*, *WNT7A*).

The establishment of early pregnancy in mammalian species, including pigs, depends on proper conceptus development and appropriate preparation of the uterine environment. Embryo implantation is one of the critical periods and is characterized by a high mortality rate in mammalian species, reaching even approximately 30–40% for porcine conceptuses^17^. Present study, is the first characterizing the gene expression profiles of *PROK1* and *PROKR1* in porcine conceptuses/trophoblasts and elucidated the mechanism of PROK1 action on trophoblast cells during the implantation and early placentation period (Figs. 7 and 8).

We demonstrated, that the expression of *PROK1* mRNA increased gradually during conceptus/trophoblast development and reached the highest levels at the onset of placentation. This finding corresponds to studies performed in human indicating that *PROK1* mRNA expression elevates gradually and peaks at 8–11 weeks of gestation^11^. In the murine placenta, highest levels of *PROK1* mRNA were observed during early pregnancy (days 9.5 and 10.5) when compared with late gestation (days 14.5 and 17.5)^15^. In contrast to studies in mice and humans, *PROKR1* mRNA expression in porcine conceptuses remained constant during the studied stages of pregnancy. The upregulation of *PROK1* mRNA abundance in porcine trophoblasts during implantation and the early placentation period corresponds with an increased expression of *PROK1* and its receptor in the endometrium^7^, which implies that PROK1 may be an important factor during conceptus development and pregnancy establishment.

Proper communication between the conceptus and uterus is essential for successful pregnancy establishment and development. It has been shown that PROK1 regulates the expression of numerous pregnancy-related genes in the human and porcine endometrium^8,18,19^; however, there is no information regarding such mechanism in trophoblast cells. Herein, we demonstrated that PROK1, acting through PROKR1 in the porcine trophoblast, stimulated the expression of genes involved in angiogenesis, immunological response, trophoblast cell adhesion, invasion, and proliferation, in addition to activating signaling pathways related to cell adhesion and proliferation. Moreover, the results from the Ingenuity Pathway Analysis linked the PROK1-affected expression of genes/proteins to processes and functions such as cell attachment, migration and invasion, cell–cell contact, cell viability, organismal death, apoptosis, the differentiation of epithelial tissue, MAPKKK cascade, release of Ca^2+^, angiogenesis, vasculogenesis, inflammatory response, the recruitment of blood cells, chemotaxis, and the synthesis of lipids. These results indicate a pleiotropic role for PROK1 in trophoblast functions. It has been well documented that gene and/or protein expressions of PROK1-regulated factors such as FGF2^20^, *IFNG*^21^, IGF1^22^, *IL1B*^23,24^, *MMP9*^25^, TBFB3^24^, *WNT4*^26^, *VEGFA,* and *FLT1*^27^ are elevated in porcine trophoblasts during implantation and early pregnancy. Moreover, other PROK1-induced genes or proteins determined in the present study – namely, *ANGPT1*, and *ANGPT2*^28^, *BGN*^25^, CDH1^26^, MAPK1/MAPK3, and PTK2^29^, *IL6*, *LIF*, *LIFR*^30^, SPP1^5,31^, *TNF*^32^, KDR^27,32^, *WNT5A,* and *WNT7A*^26^ have been shown to be expressed and play important roles in porcine trophoblasts or placenta during early pregnancy. In present study, we demonstrated that the expression of *BGN*, *IL11* and *SPP1* mRNA increased gradually during conceptus/trophoblast development and reached the highest levels at the onset of placentation. Moreover, the present findings indicated that the expression of *FGF9* increased since the beginning of implantation, and remained higher at the onset of placentation when compared to the preimplantation stage.

In mammalian species, the placenta is one of the most vascularized organs in the whole organism. In the formation of the capillary network in the placenta, one of the most important processes is angiogenesis, i.e., the process of new blood vessel formation from pre-existing blood vessels. Angiogenesis is tightly controlled by numerous angiogenic regulators, the most common of which are VEGFA, FGF2, ANGPT1, and ANGPT2^33^; however, recent studies have shown an emerging role of PROK1 during pregnancy^7–9,12^. Our previous study has demonstrated that PROK1 is involved in endometrial angiogenesis by the stimulating endothelial cell proliferation and network formation and by increasing the secretion of VEGFA and PGF2α^7^. In the present study, we showed that PROK1, acting through PROKR1 in trophoblast cells, regulated the gene expression of several angiogenic factors and their receptors, including *ANGPT1*, *ANGPT2*, *CCN2*, *CDH13*, *FGF2*, *FLT1*, *KDR*, *NFATC2*, *RCAN1*, and *VEGFA*. These findings are in agreement with our previous results indicating that PROK1 increases *ANGPT1*, *ANGPT2*, *CCN2*, *CDH13*, and *NFATC2* mRNA expression in the porcine endometrium^8^. FGF2 and VEGFA and its receptors are well known to play an important role in peri-implantation vascular events and conceptus development in pigs^27,34^. During angiogenesis angiopoietins, CCN2, CDH13, FGF2, NFATC2, and RCAN1 acting together with VEGFA and their relevant receptors, are involved in endothelial cell survival, migration, adhesion, and vessel remodeling^27,33–37^. The aforementioned studies and the findings of the present study confirm our assumption that PROK1–PROKR1 signaling can participate in angiogenesis during placenta development. Moreover, the PROK1-induced expression of *ANGPT1*, *ANGPT2*, *CCN2*, *FGF2*, *FLT1*, *KDR* and *VEGFA* may be involved in the regulation of trophoblast cell proliferation, migration, and invasion^38–42^.

Successful pregnancy establishment and development requires a precise immunological balance between the conceptus carrying paternal antigens and the maternal immune system. The modulation of the immune system prevents conceptus rejection by the maternal organism and is vital for conceptus growth, survival, and implantation^4^. Moreover, during early pregnancy in all placental mammals, including pigs, there is an extensive recruitment of lymphocytes, particularly natural killer cells, dendritic cells, and macrophages at the maternal–fetal interface^43^. Therefore, implantation is associated with signs of inflammation and increased expression of inflammatory mediators such as cytokines, growth factors, and lipids^3^. In the present study, we showed that PROK1–PROKR1 signaling increased the gene expression of cytokines *IFNG*, *IL1B*, *IL6*, *IL11*, *LIF*, *LIFR*, and *TNF* in trophoblast. Our findings are consistent with previous reports, demonstrating that PROK1 increases *IFNG*, *IL6*, *LIF*, *LIFR*, and *TNF* mRNA expression in the porcine endometrium^8^. Prokineticin 1-stimulated cytokines such as IL1B, TNF and cytokines belonging to the IL 6 family (IL6, IL11, and LIF), together with their receptors, are important for pregnancy establishment and placentation^23,32,44,45^. These cytokines are involved in trophectoderm remodeling, proliferation, attachment to the endometrium, and the regulation of trophoblast cell migration and invasion^1,30,38,44^. Moreover, during implantation, IL1B and IFNG play important roles in the regulation of immunotolerance at the maternal–fetal interface^34,46^. In pigs, IFNG stimulates endometrial expression of IFN-regulated genes that are involved in the release of histotroph, cell proliferation, cell development, and attachment of the conceptus^46^. The present results confirmed our hypothesis that PROK1–PROKR1 signaling contributes to the regulation of immune response and the creation of proinflammatory environment in uterus by increasing the expression of cytokines in the porcine trophoblast during attachment and early placentation.

Proper conceptus development and placentation also depend on cell differentiation, proliferation, adhesion, migration, and invasion. The present study reveals that PROK1 increases the proliferation of porcine trophoblast cells on days 15 and 20 of pregnancy and stimulates their adhesion to the ECM. Moreover, using a specific PROKR1 antagonist, we demonstrated that the stimulatory effects of PROK1 on conceptus cell proliferation and adhesion depended on PROK1–PROKR1 signaling. Our results are in agreement with previous studies using BeWo and JEG-3 choriocarcinoma lines suggesting that PROK1 promotes human trophoblast cell proliferation^13^ and adhesion^19^. Using specific inhibitors, we demonstrated that PROK1–PROKR1 signaling elevated trophoblast cell proliferation by activating MAPK, PI3K/AKT/mTOR kinases, and cAMP pathways on days 15 and 20 of pregnancy. Trophoblast cell adhesion was induced by activation of MAPK (on days 15 and 20 of pregnancy) and PI3K/AKT signaling pathways (on day 20 of pregnancy). We also indicated that in the porcine trophoblast cells PROK1 induced MAPK1/3 and PTK2 phosphorylation and stimulated the expression of genes involved in the regulation of trophoblast cell adhesion, invasion, and proliferation, such as *BGN*, *CDH1*, *FGF9*, *IGF1*, *MMP9*, *MUC4*, *SPP1*, *TGFB3*, *WNT4*, *WNT5A*, and *WNT7A*. Our results are consistent with previous studies indicating that PROK1 increases MAPK1/3 phosphorylation in the BeWo choriocarcinoma cell line^13^. Moreover, the present results are in agreement with our previous findings revealing that PROK1 stimulates MAPK and PTK2 phosphorylation and *FGF9*, *MUC4*, *SPP1*, and *TGFB3* gene expression in the porcine endometrium^7,8^.

PROK1 induced the trophoblast expression of the genes *BGN*, *CDH1*, *FGF9*, *IGF1*, *MMP9*, *MUC4*, *SPP1*, *TGFB3*, *WNT4*, *WNT5A*, and *WNT7A,* which are implicated to participate in regulating processes such as cell differentiation, proliferation, adhesion, migration, or invasion during conceptus development and implantation^1,4–6,31,38,47,48^. In addition, in humans, TGFB3, CDH1, and WNT5A are involved in the inhibition of trophoblast invasiveness^49–51^. Moreover, in the porcine endometrium, *MUC4* gene expression is upregulated during early pregnancy and is supposed to be responsible for modulating the proteolytic capacity of the conceptus and protecting the LE from trophoblast invasion^52^. It can be suggested that the regulation of *TGFB3, CDH1, MUC4,* and *WNT5A* expression via PROK1 in trophoblasts may be related to the non-invasive type of placentation in pigs. Another important gene induced by PROK1 is *MMP9*, which has been demonstrated to regulate invasion and the migration of human trophoblast cells^53^. Our previous studies show evidence that PGF2α stimulates *MMP9* mRNA expression in the porcine endometrium, suggesting its involvement in ECM remodeling^54^. In porcine conceptuses, *MMP9* gene expression increases gradually and reaches the highest level on days 20–25 of pregnancy. This in turn suggests the participation of MMP9 in trophoblast ECM remodeling during placentation. Secreted phosphoprotein 1 binds to cell surface integrins that promote cell–cell and cell–ECM adhesion which further leads to cytoskeletal reorganization and the stabilization of adhesion^1^. The present study indicated that expression of *SPP1* mRNA in porcine conceptuses increased gradually and reached the highest level on days 20–25 of pregnancy. Interestingly, SPP1 has also been reported to regulate the fetal/placental development in pigs and has been suggested to play an important role in conceptus attachment and placentation^31^. The aforementioned findings, together with our present results, confirm our assumption that PROK1, acting by PROKR1, can stimulate the proliferation and adhesion of porcine trophoblast cells and regulate trophoblast cell invasiveness. In addition, it may play an important role in conceptus development.

In conclusion, the present study provides evidence for the important pleiotropic role of PROK1 in trophoblast function during implantation and early placentation. For the first time, we have demonstrated that conceptus/trophoblast expression of *PROK1* mRNA increases gradually and reaches its highest level on days 20–25 of pregnancy (beginning at placentation) in pigs. Our study indicates that PROK1, acting by PROKR1, regulates the expression of the pregnancy-related genes *ANGPT1, ANGPT2, CCN2, CDH13, FGF2, FLT1, KDR, NFATC2, RCAN1, VEGFA, IFNG, IL1B, IL6, IL11, LIF, LIFR, TNF, BGN, CDH1, FGF9, IGF1, MMP9, MUC4, SPP1, TGFB3, WNT4, WMT5A*, and *WNT7A* which are involved in processes such as angiogenesis, immunological response, trophoblast cell adhesion, invasion, migration and proliferation, cell–cell contact, apoptosis, the differentiation of epithelial tissue, MAPKKK cascade, the release of Ca2+, angiogenesis, vasculogenesis, inflammatory response, the recruitment of blood cells, chemotaxis, and the synthesis of lipid (Figs. 7 and 8). Furthermore, PROK1, acting through PROKR1, increases the adhesion and proliferation of trophoblast cells. The PROK1-stimulated proliferation of trophoblast cells isolated on days 15 and 20 of pregnancy is mediated by the PI3K/AKT/mTOR, MAPK and cAMP signaling pathways, while PROK1-induced adhesion is mediated by the MAPK (on days 15 and 20 of pregnancy) and PI3K/AKT signaling pathways (on day 20 of pregnancy). Our study suggests PROK1-PROKR1 signaling as a new target for investigations in early pregnancy loss.

## Materials and methods

All of the procedures involving animals were approved by the Local Research Ethics Committee of University of Warmia and Mazury in Olsztyn, Poland, and were conducted in accordance with the national guidelines for agricultural animal care, and the ARRIVE guidelines.

### Experiment 1. The expression profiles of PROK1, its receptor (PROKR1), and some of the pregnancy-related genes in the porcine conceptus/trophoblast

Peripubertal crossbred gilts of similar age (6–6.5 months) and genetic background, after two natural estrous cycles were bred at the onset of estrus (day 0) then 12 h and 24 h later. Pregnant gilts were slaughtered at the local abattoir between days 10 and 25 of pregnancy. Samples were collected as described previously^29^. Based on the days of pregnancy and conceptus morphology, the trophoblast or conceptus tissues derived from the same animal were pooled and classified into groups: days 10–12 (preimplantation stage; spherical and tubular forms; *n* = 5), days 14–16 (beginning of attachment/implantation; filamentous forms, *n* = 6), days 17–19 (implantation stage, filamentous forms; *n* = 4) and days 20–25 (chorion which originates from trophoblast, the onset of placenta development; *n* = 5). Dissections of trophoblast tissues from conceptuses were performed since day 18 post mating^29^. Therefore, expression of studied genes was analyzed in 10- to 17-day conceptuses and 18- to 25-day trophoblast tissues. Collected conceptuses/trophoblasts were snap-frozen in liquid nitrogen and stored at − 80 °C. The expression profiles of *PROK1*, *PROKR1*, *BGN*, *FGF9*, *IL11*, and *SPP1* genes in collected conceptuses/trophoblasts samples were determined using real-time RT-PCR as described in the subchapter *Real-time RT-PCR*.

### Experiment 2. Effect of PROK1 on the expression of the pregnancy-related genes in the porcine trophoblast

#### Isolation and culture of porcine primary trophoblast cells

Porcine trophoblast cells were isolated as described previously^29^ from conceptuses recovered from uteri of pregnant gilts on day 15 (n = 10) and 20 of pregnancy (n = 7). To assess the purity of cell cultures, isolated cells were stained for the presence of the trophoblast marker—cytokeratin as described previously^25^. Purity of cell cultures was assessed at 98%. After isolation, cells were plated onto plates and cultured in medium M199 (M3769; Sigma-Aldrich, St. Louis, MO, USA) containing 10% of (v/v) newborn calf serum (NCS; Sigma-Aldrich) and antibiotics at 37 °C in a humidified atmosphere containing 95% air and 5% CO_2_.

#### Effect of PROK1 on the expression of pregnancy-related genes

The effect of PROK1 on the expression of selected pregnancy-related genes was studied using cultured porcine trophoblast cells collected from day 15 and 20 of pregnancy. The primary trophoblast cells were seeded at 0.5 × 10^6^ cells/well in 6-well plates. After reaching 80% confluence, the cells were incubated with medium M199 supplemented with 1% (v/v) NCS and antibiotics containing the control vehicle, 0.01% ethanol) or PROK1 (40 nM; 100–44, PeproTech, Rocky Hill, NJ) for 24 h in the presence/absence of a PROKR1 antagonist (PC7; 1 µM). The PC7 antagonist was kindly gifted by Dr. Gianfranco Balboni. The doses of PROK1 and PC7 were chosen based on previous studies^7,8^. After incubation, the cells were lysed in Fenozol buffer (A&A Biotechnology, Gdansk, Poland) and stored at − 80 °C until gene expression analyses. Details related to gene expression analyses are described in the subchapter *Real-time RT-PCR*.

### Experiment 3. The effect of PROK1 on signaling pathways and trophoblast cell adhesion and proliferation

To determine whether PROK1 may affect selected signaling pathways, adhesion and proliferation of the porcine trophoblast cells, an in vitro model was used. Porcine primary trophoblast cells on days 15 and 20 of pregnancy were isolated as described in the subchapter *Isolation and culture of porcine primary trophoblast cells* in Experiment 2.

#### Phosphorylation of MAPK1/3 and PTK2

The effects of PROK1 on PTK2, and MAPK phosphorylation were studied using porcine trophoblast cells seeded at 0.5 × 10^6^ cells/well in 6-well plates. After reaching 80% confluence, the cells were treated with medium M199 containing control (vehicle, 0.01% ethanol) or PROK1 (40 nM) for 10 min in the presence/absence of a PROKR1 antagonist (PC7; 1 µM). Following treatment, the cells were lysed in RIPA buffer [50 mM Tris–HCl (pH = 7.4), 150 mM NaCl, 1% Triton X-100, 0.5% sodium deoxycholate, 0.1% SDS and 1 mM EDTA (pH = 8.0)] and stored at -30 °C until phosphorylation analyses by Western blot as described below.

#### Adhesion assay

Confluent cells were incubated in 6-well plates (0.5 × 10^6^ cells/well) with medium M199 supplemented with 1% (v/v) NCS and antibiotics containing vehicle (0.01% ethanol) or PROK1 (40 nM) in the in the presence/absence of PC7 (1 µM), MAPK inhibitor (25 µM; PD 098059; Sigma Aldrich) or PI3K/AKT kinase inhibitor (25 µM; LY 294002; Sigma Aldrich) for 24 h at 37 °C in a humidified atmosphere containing 95% air and 5% CO_2_. After treatment, the cells were detached from the 6-well plate, and 100 μL (0.4 × 10^5^ cells/well) of each cell suspension was transferred onto Millicoat Cell Adhesion Strips coated with human fibronectin (ECM101; Millipore, Burlington, MA, USA) and incubated for 2 h at 37 °C in a humidified atmosphere containing 95% air and 5% CO_2_. Following incubation, cell adhesion was measured using a colorimetric method accordingly to the manufacturer’s protocol. Experiments were repeated seven times (for each day 15 and 20 of pregnancy) in duplicates. The fold difference was determined by dividing the absorbance displayed by PROK1-treated cells by the absorbance displayed by control-treated cells.

#### Proliferation assay

A proliferation assay was performed as described previously^7,54^ with some modifications. Briefly, trophoblast cells after reaching 40% confluence (96-well plate; 0.3 × 10^5^ cells/well) were incubated for 24 h with vehicle (0.01% ethanol) or PROK1 (40 nM) in the presence/absence of prokineticin receptor 1 antagonist (PC7; 1 µM), MAPK inhibitor (25 µM; PD 098059; Sigma Aldrich), PI3K/AKT kinase inhibitor (25 µM; LY 294002; Sigma Aldrich), AKT kinase inhibitor (2.5 µM; Sigma Aldrich), cAMP synthesis inhibitor (25 µM; SQ 22536; Sigma Aldrich) and mTOR kinase inhibitor (50 nM; rapamycin; Sigma Aldrich). After treatment, proliferation was assessed using the Cell Titer 96 Aqueous One Solution Proliferation Reagent (Promega; Madison, WI, USA). Experiments were performed in triplicates and repeated nine times or seven times (day 15 and 20 of pregnancy; respectively). The fold difference was calculated by dividing the absorbance obtained for PROK1-treated cells by the absorbance obtained for control-treated cells.

#### Real-time RT-PCR

Isolation of RNA, reverse transcription, and real-time PCR for samples from Experiment 1 and 2 were performed as described previously^8,25,54,55^. The mRNA expression of *PROKR1*, *VEGFA*, *FLT1*, *KDR*, *LIF*, *LIFR*, *WNT4*, *WNT5A* and *WNT7A* were analyzed using Power SYBR Green (Life Technologies, Carlsbad, CA, USA) with oligonucleotide primers (1 μM; Supplementary Table 1). The gene expression of *PROK1*, *ANGPT1*, *ANGPT2*, *BGN*, *CCN2*, *CDH1*, *CDH13*, *FGF2*, *FGF9*, *IFNG*, *IGF1*, *IL11*, *IL1B*, *IL6*, *MMP9*, *MUC4*, *NFATC2*, *RCAN1*, *SPP1*, *TGFB3*, and *TNF* was analyzed using the TaqMan probes (Life Technologies) according to the manufacturer’s protocol (Supplementary Table 1). Details of the PCR programs are provided in Supplementary Table 1. Gene expression values were calculated using real-time PCR Miner Software (http://ewindup.info/miner/)^56^. The stability of the reference genes (*GAPDH, ACTB,* and *PPIA*) was examined by using the statistical algorithm Norm Finder 2.0^57^. Gene expression values in conceptus/trophoblast tissues collected from pregnant gilts (days 10–25) was normalized against *PPIA* expression. Gene expression values in trophoblast cells isolated from gilts on days 15 and 20 of pregnancy after in vitro experiment was normalized against the geometric mean of *ACTB* and *PPIA*.

#### Western blot

Phosphorylation of PTK2, pPTK2, MAPK and pMAPK proteins (Experiment 3) was evaluated by Western blot analyses as described earlier^58^. Briefly, 50 μg of total protein extracts were dissolved in SDS gel loading buffer (50 mM Tris–HCl (pH 6,8), 4% SDS, 20% glycerol, and 2% β-mercaptoethanol), heated at 95 °C for 4 min, and separated on 8% Tris–Glycine SDS-PAGE. Separated proteins were electroblotted onto a 0.4 µm polyvinylidene fluoride membranes. The membranes were incubated overnight with primary antibodies (Supplementary Table 2) at 4 °C. Subsequently, the membranes were incubated with secondary horseradish peroxidase-conjugated antibodies (Supplementary Table 2) for 90 min at room temperature. Immune complexes were visualized using a Clarity™ Western ECL Substrate (Bio-Rad, Hercules, CA, USA). Protein expression was normalized against GAPDH expression.

### Experiment 4. Identifications of biofunctions enriched for PROK1-regulated genes/proteins in the porcine trophoblast using Ingenuity Pathway Analysis

To identify the signaling pathways, molecular networks, and biological functions of PROK1-regulated genes in the porcine trophoblast, Ingenuity Pathway Analysis (IPA; v. 01.12; Qiagen, Hilden, Germany) was used. Processes and functions potentially affected by PROK1 were identified using "Diseases and biofunctions" analysis and visualized in Path Designer tool. Results were summarized in graphic format.

### Statistical analyses

Statistical analyses were performed by GraphPad Prism software version 9.0 (GraphPad Software, Inc., San Diego, CA, USA). All data were tested for normality and homoscedasticity. Before using parametric tests, log-transformation of data was performed when needed. The results of Experiment 1 were analyzed by one-way analysis of variance (ANOVA), followed by the Bonferroni post-hoc test. The effects of PROK1 on the expression of the selected pregnancy-related genes (Experiment 2), the phosphorylation of MAPK 1/3 and PTK2 (Experiment 3) were evaluated using two-way ANOVA with the Bonferroni post-hoc test. The statistical significance of the effect of PROK1 on trophoblast cell proliferation and adhesion was evaluated using one-way ANOVA, followed by Bonferroni post-hoc test. *P* < 0.05 was considered statistically significant.

### Ethics approval

The materials collected were reviewed and accepted following the guidelines of the Local Ethics Committee for Experiments on Animals of University of Warmia and Mazury in Olsztyn, Poland, and conducted in accordance with the national guidelines for agricultural animal care, and the ARRIVE guidelines.

## Supplementary Information

Supplementary Information 1.

Supplementary Table 3.

## Data Availability

The datasets used and/or analyzed during the current study are available from the corresponding author on reasonable request.
