# Supplementary material for: Prokineticin 1–prokineticin receptor 1 signaling in trophoblast promotes embryo implantation and placenta development
Source: Sci Rep. 2021 Jul 2;11:13715. doi: 10.1038/s41598-021-93102-1 (PMC8253840; doi:10.1038/s41598-021-93102-1)
Supplement: Supplementary file 1 — Supplementary Information 1. [file 41598_2021_93102_MOESM1_ESM.pdf]

Supplementary Information

Prokineticin 1–prokineticin receptor 1 signaling in trophoblast promotes embryo implantation and placenta development

Ewelina Goryszewska[1], Monika Baryla[1], Piotr Kaczynski[1], and Agnieszka Waclawik[1*]

[1] Institute of Animal Reproduction and Food Research of the Polish Academy of Sciences, Tuwima 10, 10-748 Olsztyn, Poland.

Supplementary data summary:

Supplementary Figure 1. Uncropped Western blot images displayed in Figure 6A for expression of: mitogen-activated protein kinase (MAPK1/3) (A, B); phosphorylated MAPK1/3 (pMAPK1/3) (C, D); glyceraldehyde-3-phosphate dehydrogenase (GAPDH) (E, F) in samples from days 15 (A, C and E) and 20 of pregnancy (B, D and F). (E) and (F) Western blots were reprobed with GAPDH antibodies. The dotted rectangles delineate the areas shown in Fig. 6A.

Supplementary Figure 2. Uncropped Western blot images displayed in Figure 6B for expression of: protein tyrosine kinase 2 (PTK2) (A, B); phosphorylated PTK2 (pPTK2) (C, D); glyceraldehyde-3-phosphate dehydrogenase (GAPDH) (E, F) in samples from days 15 (A, C and E) and 20 of pregnancy (B, D and F). (E) and (F) Western blots were reprobed with GAPDH antibodies. The dotted rectangles delineate the areas shown in Fig. 6B.

Supplementary Table 1. Primer sequences and assays used in real-time RT-PCR analyses and condition of real-time RT-PCR.

Supplementary Table 2. List of antibodies used in Western blot experiments.

Supplementary Table 3. List of selected (z-score above 1.7) processes and functions potentially affected by PROK1 obtained by diseases and biofunctions analysis in Ingenuity Pathway Analysis Software. *Separate Microsoft Excel file attached to this article.*

Supplementary Figure 1

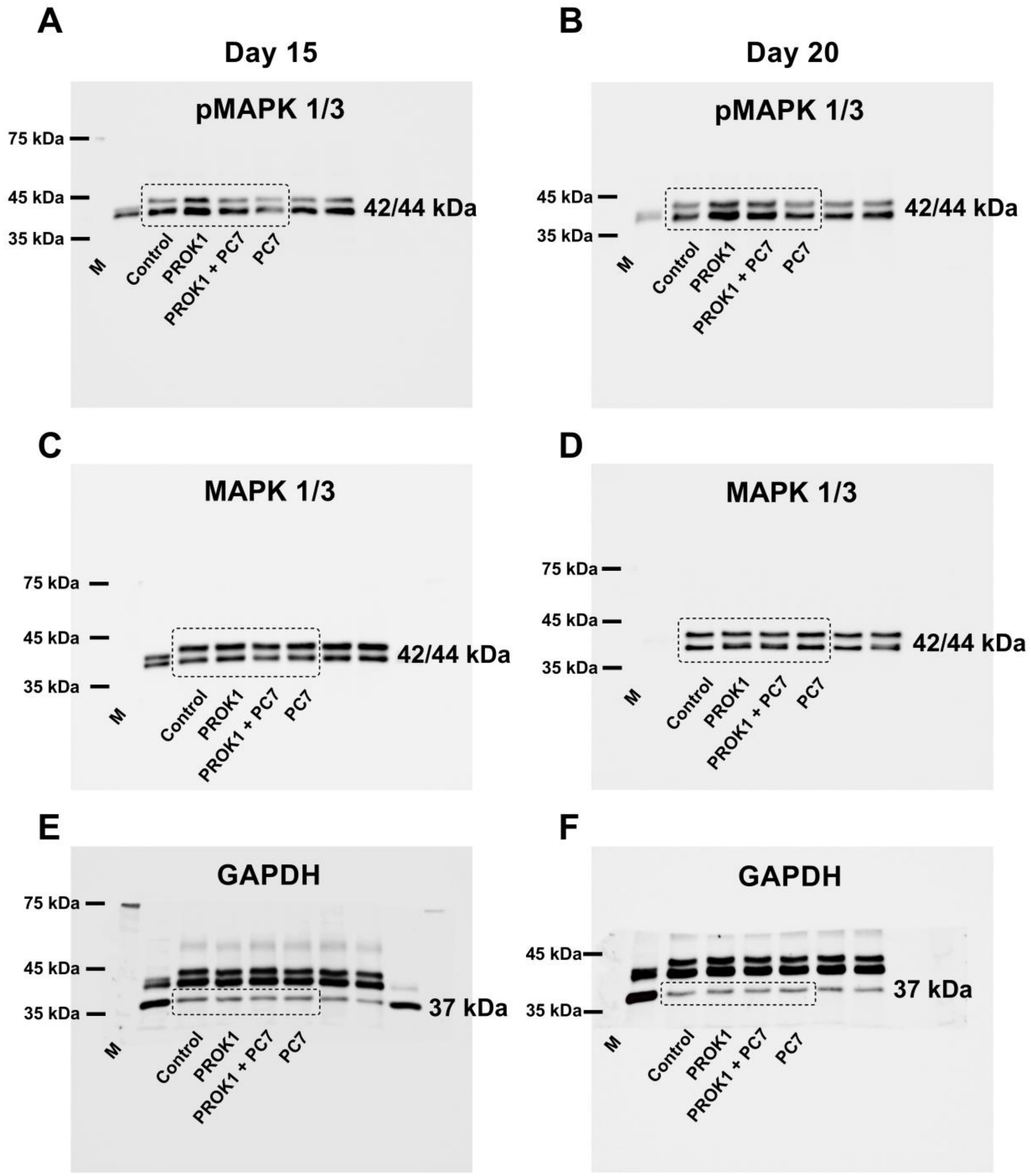

Supplementary Figure 1. Uncropped Western blot images displayed in Figure 6A for expression of: mitogen-activated protein kinase (MAPK1/3) (A, B); phosphorylated MAPK1/3 (pMAPK1/3) (C, D); glyceraldehyde-3-phosphate dehydrogenase (GAPDH) (E, F) in samples from days 15 (A, C and E) and 20 of pregnancy (B, D and F). (E) and (F) Western blots were reprobed with GAPDH antibodies. The dotted rectangles delineate the areas shown in Fig. 6A.

Supplementary Figure 2

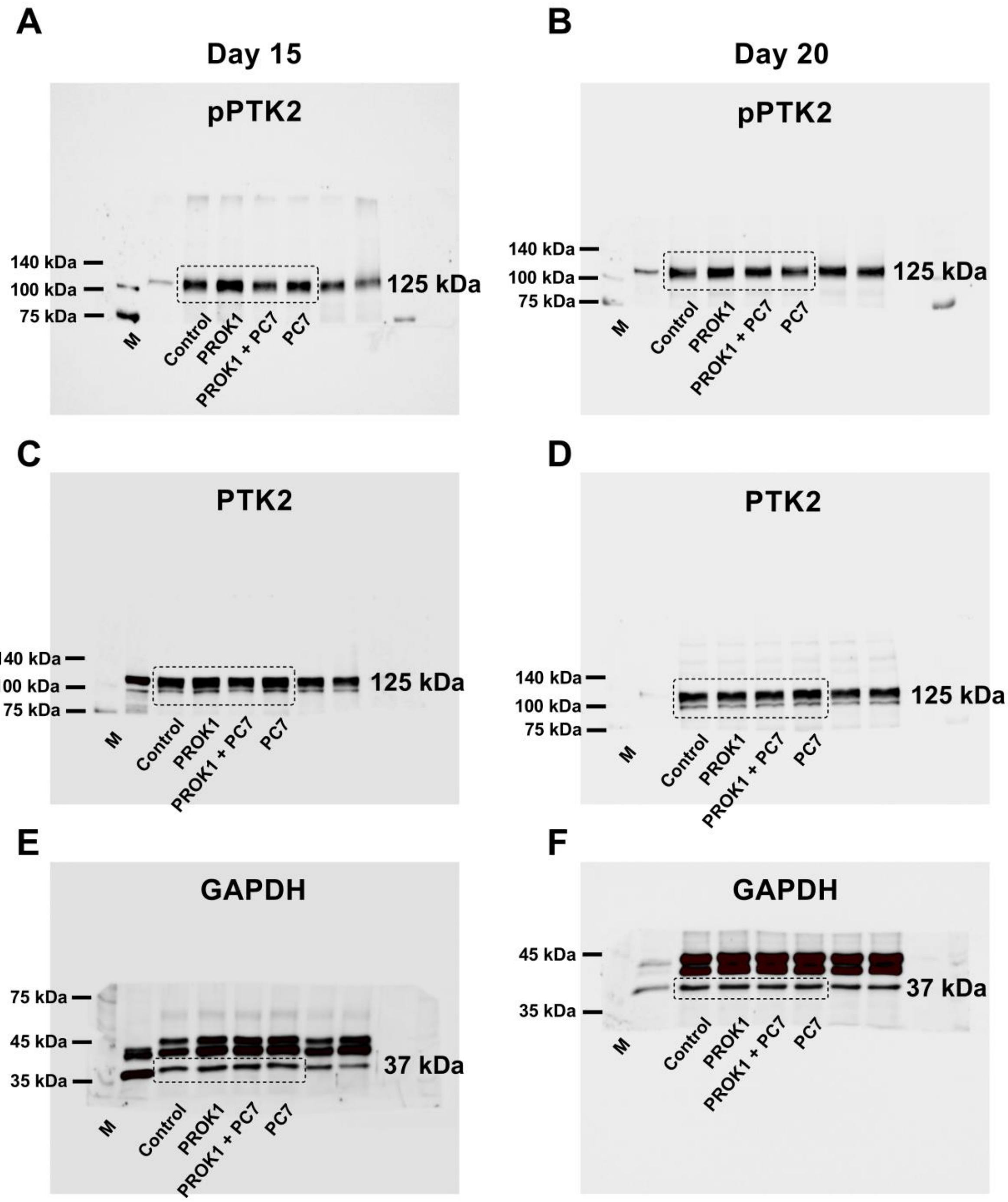

Supplementary Figure 2. Uncropped Western blot images displayed in Figure 6B for expression of: protein tyrosine kinase 2 (PTK2) (A, B); phosphorylated PTK2 (pPTK2) (C, D); glyceraldehyde-3-phosphate dehydrogenase (GAPDH) (E, F) in samples from days 15 (A, C and E) and 20 of pregnancy (B, D and F). (E) and (F) Western blots were reprobed with GAPDH antibodies. The dotted rectangles delineate the areas shown in Fig. 6B.

Supplementary Table 1. Primer sequences and assays used in real-time RT-PCR analyses and condition of real-time RT-PCR.

Gene	Number of TaqMan probes or Primers sequences (5’-3’)	GenBank accession no.	Conditions °C, time
PROK1	Ss04246562_m1	NM_001172586.1	40 cycles of: denaturation (15 s at 95°C) and annealing and elongation (60 s at 60°C)
ANGPT1	Ss03380513_u1	NM_001044573.2	
ANGPT2	Ss03392365_m1	NM_213808.1	
FGF2	Ss03375809_u1	AJ577089.1	
NFATC2	Ss03373287_m1	NM_001113452.1	
RCAN1	Ss04325819_m1	NM_001243913.1	
FGF9	Ss03381521_s1	NM_213801.1	
IGF1	Ss03394499_m1	NM_214256.1	
TGFB3	Ss03394352_m1	NM_214198.1	
CCN2	Ss03392396_g1	NM_213833.1	
CDH1	Ss03377287_u1	EF530350.1	
CDH13	Ss03386756_u1	NM_001109945.1	
MUC4	Ss04321839_g1	NM_001206344.1	
SPP1	Ss03391321_m1	NM_214023.1	
BGN	Ss03375454_u1	AF159382.1	
MMP9	Ss03392100_m1	NM_001038004.1	
IL6	Ss03384604_u1	NM_214399.1	
IFNG	Ss03391052_m1	NM_213948.1	
TNF	Ss03391316_g1	NM_214022.1	
IL1B	Ss03393804_m1	NM_214055.1	
IL11	SsARWCWNH	AB198667	
GAPDH	Ss03375435_u1	NM_001206359.1	
ACTB	Ss03376081_u1	AK237086.1	
PPIA	Ss03394782_g1	NM_214353.1	
PROKR1	Sense: 5’-GGACTTGGCACTCTCGTCTC-3’<br>Antisense: 5′-GAACAGCTCCTCCTCTTCA-3′	XM_003481198.1	36 cycles of: denaturation (15 s at 95°C) and annealing and elongation (60 s at 60°C)
FLT1	Sense: 5’-CACCCCGGAAATCTATCAGATC-3’<br>Antisense: 5′-GAGTACGTGAAGCCGCTGTTG-3’	XM_001925740	
KDR	Sense: 5’- GATGCTCGCCTCCCTTTGA-3’<br>Antisense: 5′-AGTTCCTTCTTTCAGTCGCCTACA	XM_003128987	
LIF	Sense: 5’-GAGCCATTTCCCAACAACTTG-3’<br>Antisense: 5′-TAGGCGATGATGCGGTACAG-3’	NM_214402.2	
LIFR	Sense: 5’-GCAGGAAGCAAACTAGAGATTACAG-3’<br>Antisense: 5′-ACAGCAACATCACCTCCAAAT-3’	AJ656748	
VEGFA	Sense: 5′-GAGGCAAGAAAATCCCTGTG-3′<br>Antisense: 5′-TCACATCTGCAAGTACGTTCG-3′	NM_214084	
ACTB	Sense: 5′-ACATCAAGGAGAAGCTCTGCTACG-3′<br>Antisense: 5′-GAGGGGCGATGATCTTGATCTTCA-3′	U07786	
PPIA	Sense: 5′-TAACCCCACCGTCTTCTT-3′<br>Antisense: 5′-TGCCATCCAACCACTCAG-3′	AY266299.1	
WNT4	Sense: 5’-GCATGTCCTGCTCGCAGAA-3’<br>Antisense: 5′-ACGCACTCAAGGAGAAGTTTGAC-3’	NM_001170828	36 cycles of: denaturation (15 s at 95°C), annealing (30 s at 59°C) and elongation (60 s at 72°C)
WNT5A	Sense: 5’-TCTCCTTCGCCCAGGTTGTA-3’<br>Antisense: 5′-GGCTGTGCTCCTATGATATATACTTC-3’	XM_005669658	
WNT7A	Sense: 5’-TAACGAGGCAGGCCGAAAG-3’<br>Antisense: 5′-AGTGTGGTCCAGCAGGTCTTG-3’	XM_003132396	
GAPDH	Sense: 5′-CAGCAATGCCTCCTGTACCA-3′<br>Antisense: 5′-GATGCCGAAGTTGTCATGGA-3′	AF017079	36 cycles of: denaturation (15 s at 95°C), annealing (30 s at 55°C) and elongation (60 s at 72°C)

Supplementary Table 2. List of antibodies used in Western blot experiments.

Protein Target	Name of antibody	Manufacturer, catalog no., or name of source	Species raised in monoclonal or polyclonal	Dilution Used
PTK2	FAK Antibody	Cell Signaling Technology, 3285	Rabbit, polyclonal	1:300
PhosphoPTK2	Phospho-FAK (Tyr397) Antibody	Cell Signaling Technology, 3283	Rabbit, polyclonal	1:300
MAPK1/3	P44/42 MAPK (Erk1/2) antibody	Cell Signaling, 9102S	Rabbit, polyclonal	1:300
PhosphoMAPK1/3	Phospho-p44/42 MAPK (Erk1/2) Thr202/Tyr204) antibody	Cell Signaling, 9101	Rabbit, polyclonal	1:300
GAPDH	Anti-GAPDH antibody	Abcam, ab9485	Rabbit, polyclonal	1:2000
ACTB	Anti-β-actin antibody	Abcam, ab8227	Rabbit, polyclonal	1:2000
anti-rabbit HRP	Immun-Star™ Goat AntiRabit (GAR)-HRP Conjugate	Bio-Rad Laboratories, 1705046	Goat, polyclonal	1:20 000
